# Flushing and Locking of Venous Catheters: Available Evidence and Evidence Deficit

**DOI:** 10.1155/2015/985686

**Published:** 2015-05-14

**Authors:** Godelieve Alice Goossens

**Affiliations:** ^1^Nursing Centre of Excellence, University Hospitals Leuven, 3000 Leuven, Belgium; ^2^Department of Public Health and Primary Care, KU Leuven, 3000 Leuven, Belgium

## Abstract

Flushing and locking of intravenous catheters are thought to be essential in the prevention of occlusion. The clinical sign of an occlusion is catheter malfunction and flushing is strongly recommended to ensure a well-functioning catheter. Therefore fluid dynamics, flushing techniques, and sufficient flushing volumes are important matters in adequate flushing in all catheter types. If a catheter is not in use, it is locked. For years, it has been thought that the catheter has to be filled with an anticoagulant to prevent catheter occlusion. Heparin has played a key role in locking venous catheters. However, the high number of risks associated with heparin forces us to look for alternatives. A long time ago, 0.9% sodium chloride was already introduced as locking solution in peripheral cannulas. More recently, a 0.9% sodium chloride lock has also been investigated in other types of catheters. Thrombolytic agents have also been studied as a locking solution because their antithrombotic effect was suggested as superior to heparin. Other catheter lock solutions focus on the anti-infective properties of the locks such as antibiotics and chelating agents. Still, the most effective locking solution will depend on the catheter type and the patient's condition.

## 1. Introduction

**Figure 1:**
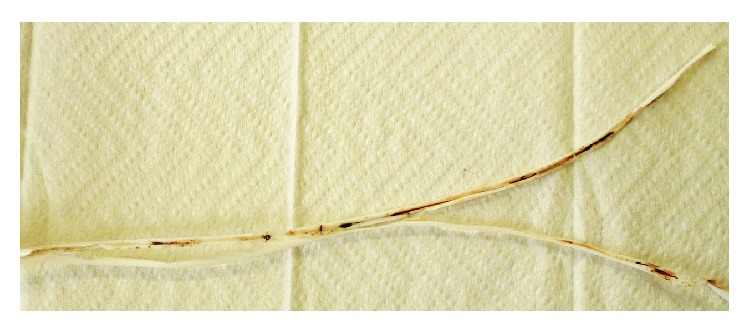
Visible adhesions to the catheter wall.

**Figure 2:**
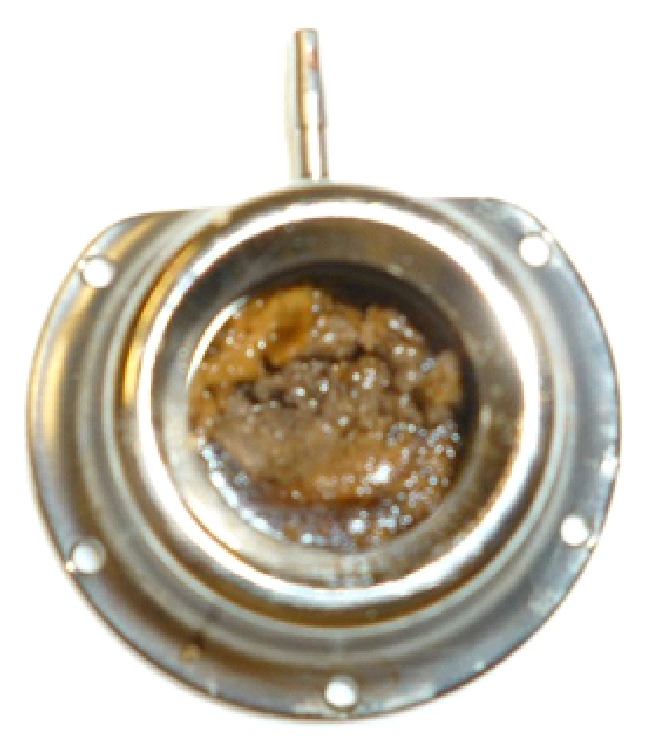
Build-up of deposits of fibrin and/or infusion fluids and/or drug precipitates.

Flushing and locking have been strongly associated with the prevention of catheter occlusion. The causes of catheter occlusion might be thrombotic, related to drug or parenteral nutrition (PN) precipitates or mechanical. Thrombotic obstruction is caused by an intraluminal clot or a catheter tip thrombus. Precipitates might be formed by drug mixtures with an extreme pH, calcium phosphate crystals, or lipid deposits. Examples of mechanical obstruction are sleeve formation resulting in partial or total embedding of the catheter tip, a catheter tip abutting the vein wall, a pinch off, a kinked or twisted catheter or tubing, tight sutures, or an incorrect Huber needle placement [1]. However these mechanical occlusions are extraluminal causes of obstruction. Flushing and locking maneuvers will not impact these types of occlusion. On the contrary flushing and locking are strongly associated with intraluminal occlusion following build-up of deposits of fibrin and/or infusion fluids (like PN and dextrose) or a mixture of incompatible medications and solutions (Figures 1 and 2). Adequate flushing and locking might also eliminate all potential nesting material for microorganisms and thus also reduce the risk of catheter-related bloodstream infection (CRBSI) [2].

The aim of this paper is to clarify issues related to flushing and locking and to describe the available evidence relating to the benefits of interventions in relation to occlusion. All types of intravenous (IV) catheters are considered apart from apheresis and haemodialysis catheters and catheters in neonates due to the specific context of these devices.

## 2. Definition

In this context of rinsing the catheter, flushing of an IV catheter is defined as a manual injection of 0.9% sodium chloride or so called normal saline (NS) in order to clean the catheter. Locking is defined as the injection of a limited volume of a liquid following the catheter flush, for the period of time when the catheter is not used, to prevent intraluminal clot formation and/or catheter colonization. Traditionally, an anticoagulant, such as diluted heparin, is used. Generally, flushing and locking are described ambiguously in guidelines and in the scientific literature which leads to confusion and misunderstanding. Moreover, flushing and locking are terms that are mutually exchanged [3–5]. The clinical sign of occlusion is malfunction. Catheter malfunction is any condition where, at least, injection or aspiration is no longer easy but has become difficult or impossible [6].

## 3. Flushing

### 3.1. Flushing Technique

Important aspects related to flushing are syringe diameter and injection flow dynamics. Traditionally, syringes with at least a diameter of 10 mL are recommended for long-term central venous catheters. However, this issue arises only when force applied meets resistance. Flushing with a small syringe diameter or with high force applied to the plunger in cases of resistance increases the risk of catheter damage [7]. This is particularly true in silicone rubber catheters like tunnelled catheters which have a lower material strength than polyurethane ones [8]. In these types of catheters, weak spots originate when catheters are unintendedly stretched, especially in children. Subsequently, even an injection with a 10 mL-diameter syringe may result in a catheter rupture. In contrast, most peripherally inserted central catheters (PICCs) are made of a polyurethane sort of material and some are even approved for the high pressure of CT-power injection. Also, almost all totally implantable venous access devices (TIVADs) or so called ports, in the marketplace, are power-injectable nowadays [9]. The strict need to use only a minimum of 10 mL-diameter syringes is redundant if these catheters and ports may withstand the high pressure of power injection.

The dynamic of the injection flow plays a pivotal role in adequate flushing. Vigier and colleagues showed in a qualitative* in vitro* study that flushing with an unsteady flow resulted in a significant reduction of the time scale of deadhesion of solid deposits compared to flushing with a laminar flow [10]. This research confirms the promoted practice of using a so-called push-pause, pulsatile, or turbulent technique to enhance the rinsing effect in the catheter. Furthermore, based on physics, not only the flow type but also the time interval between two boluses is critical for efficient flushing. Indeed, Guiffant and colleagues filled a catheter lumen with a protein based liquid albumin in a laboratory setting. Ten mL of NS was injected under two experimental conditions for catheter flushing, a laminar, and a pulsed flow. They measured the amount of recovered albumin from the lumen of the tested devices. They found that intermittent flushes of 10 times one mL boluses with a time interval of 0.4 s between two boluses was more efficient to rinse the catheter than shorter or longer pauses between two boluses. A continuous low flow infusion (500 mL/24 h) was the less efficient [11]. Therefore, following IV therapy, even after a continuous infusion of a 1000 mL of NS, a manual flush of 10 mL is recommended. No RCT was found which investigated the effectiveness of this intermittent flush versus a laminar injection flow technique.

### 3.2. Flushing Volume

An adequate flush volume is needed to be able to remove debris and fibrin deposits in the catheter and port reservoir. Recommendations state the following: “use at least twice the volume of the catheter and add-on devices” [3], and then the controversial words follow, “usually 5–10 mL” [4]. It is clear that 5–10 mL is a much higher volume than twice the catheter volume. However, especially in long catheters such as PICCs and tunnelled catheters a larger volume than 5 mL might be necessary to rinse the catheter. This is also the case in TIVADs because TIVADs consist of a catheter and a port reservoir. The reservoir has a dead space and a larger inner volume than a standard catheter. Adherence of lipid, fibrin, and other drug deposits to the reservoir wall may result in colonization of microorganisms and subsequently in CRBSI. Therefore in TIVADs, culturing the reservoir is more sensitive than the catheter tip if port-related infection is suspected [12, 13]. Furthermore, inadequate flushing might result in debris accumulation in the reservoir, so called sludge [14]. Clearing the chamber requires a sufficient flushing volume which may vary depending on the flow rate and the port type [15]. Ten mL of NS is commonly assumed as an adequate flushing volume in IV catheters. However, Guiffant and colleagues found in their* in vitro* study that even after a pulsatile flush with 10 mL a 100% removal of the proteins was not obtained [11]. In particular viscous products are more difficult to remove from the catheter wall. Indeed, a higher risk of early catheter-related infection was found when blood products and PN were administrated through long-term IV catheters [16]. Based on these findings a flush volume of 20 mL is suggested after infusion of viscous products such as blood components, PN, and contrast media. Unfortunately, clinical studies with different flushing volumes are lacking.

### 3.3. Flushing Regimen

Flushing the catheter is the most important factor in preventing malfunction by maintaining catheter patency. The fact that fibrin and other deposits are impeded in attaching to the intraluminal catheter wall is paramount. Therefore a major recommendation is to flush before and after administration of medication, also known as the SAS acronym. The order of IV injections is as follows: a normal saline flush (S), followed by the administration (A) of drugs or fluids, followed by a normal saline flush (S). The use of the similar sequence is even more important for blood sampling procedures due to the viscous nature of blood: SBS, a normal saline flush (S), followed by the blood sampling (B), followed by a normal saline flush (S). If the procedure ends with a heparin (H) lock the acronym is SASH and SBSH. The first NS flush provides a clean intraluminal surface which precludes attachment of drug deposits or fibrin. The flush at the end of the IV administration or blood sampling procedure prevents accumulation by intraluminal drug deposits or fibrin and a clean surface impedes attachment from microorganisms to the inner wall. A 10 mL flushing volume after blood sampling is appropriate because fibrin contact with the catheter wall is limited to some minutes. In contrast, after a blood transfusion a flush of 20 mL is required because fibrin might deposit to the catheter wall during a prolonged time. Similarly, accidental blood reflux into the catheter and infusion line, for example, when a infusion bag is empty, requires a manual flush of at least 10 mL of NS.

Flushing recommendations that are based on research and insights are summarized in Table 1.

## 4. Locking

The goal of an adequate catheter lock is prevention of premature termination of catheter function by maintaining patency when the catheter is not in use. The optimal lock solution prevents clot formation in the catheter and at the catheter tip, and also prevents microorganism adhesion and biofilm formation.

### 4.1. Locking Technique

As far back as in 1987, Shearer suggested using the positive pressure technique to prevent backflow of blood into the catheter. This technique was defined as withdrawing the syringe from the injection site while still exerting pressure on the syringe plunger when injecting the last 0.5 mL [17]. Alternatively, this could be prevented by clamping the catheter while injecting the last 0.5 mL. Nowadays, technologies may replace this manual positive pressure technique such as specially designed syringes with a plunger rod design (e.g., BD PosiFlush prefilled saline syringe), neutral or positive displacement connectors, or valves integrated in catheters (e.g., Groshong catheter, C.R. Bard).

Although the idea of preventing blood influx at the catheter tip by the positive pressure technique is reasonable, some issues arise. This technique prevents only blood influx at the moment of locking of the catheter. Once the syringe is removed, other effects might influence the internal volume such as the clamp that might be opened and closed or external catheter parts that might be pinched. This phenomenon causes a push out of locking solution and once the pressure of the pinching/clamping is lifted, the same volume that has been pushed out will create a backflow of blood at the catheter tip by negative pressure. From* in vitro* studies we know that this pinching also occurs with arm movements in long catheters inserted in an arm vein. Abduction of the arm will create a larger catheter volume and generates influx at the catheter tip. On the contrary, adduction of the arm will result in a smaller catheter volume and a displacement of the locking volume. Therefore the authors suggest choosing catheter material that minimises variation in catheter volume [18]. Iterative movement of locking volume and blood at the catheter tip is assumed in catheters inserted in the arm or catheters with an external part that might be pinched. This is especially the case in silicone catheters. On the contrary, silicone catheters have a smaller internal/outer diameter ratio whereby the volume of displacement will be smaller than with polyurethane catheters. However the clinical implications of this phenomenon are lacking; in other words it is unclear if this will lead to a higher rate of catheter occlusion and infection. The same phenomenon is observed with increased intrathoracic pressures in cases of, for example, vomiting, coughing, and crying. And, in TIVADs, blood influx will be present when the Huber needle is removed because the port septum is slightly lifted. This lifting creates a small influx of blood at the catheter tip. When the needle leaves the septum, the septum returns to its normal position and. again, it produces a small positive displacement of locking solution [19]. Once more, the clinical relevance of these fluids movements at the catheter tip is unknown. Moreover, it is likely that the use of a heparin lock before needle removal does not have an added value in, for example, a lower incidence of malfunction problems. Indeed, in a study on locking TIVADs with NS or with heparin, the Huber needle was removed without exerting positive pressure to overcome the blood influx. No more malfunction was found in the NS group compared to the heparin group [20]. No studies which focused on the malfunction rate with versus without the use of the manual positive pressure technique (without any help of connectors or valved catheters) have been found.

### 4.2. Locking Volume

The locking volume must be sufficient to fill the entire catheter. Therefore the volume of add-ons might be added to the priming volume of the catheter. Internal catheter volumes are relatively small: approximately 0.03 mL for a peripheral catheter, 0.4 mL for a 4 Fr midline, 0.6 mL for a 4 Fr single lumen nontunnelled central venous catheter (CVC), 0.7 mL for a 4 Fr PICC, 0.7 mL, and 1.5 mL for a small and large bore tunnelled catheter (75 cm), respectively, and 1.3 mL for a TIVAD (large reservoir volume of 0.5 mL), Huber needle with extension set included.

For trimmed catheters with a circular diameter the catheter volume might be calculated easily per cm. The mathematic formula of a cylinder is pi × *r* ^2^ × *h*, whereby “*r*” represents the radius (or half of the diameter) and “*h*” the height, in our case, the length of the catheter. It is clear from this formula that the catheter diameter plays a more dominant role than the catheter length.  Table 2 tabulates the volumes expressed for different internal diameters (ID) of single lumen catheters. A few examples of available catheters made of different materials are presented in Table 3. Volumes can be easily used to calculate the approximate volume of a trimmed catheter. Note that a difference in catheter length of 10 cm does not result in a substantial extra lock volume: 0.02 mL for a catheter with an ID of 0.5 mm (e.g., a single lumen Hickman 2.7 Fr) and 0.2 mL for a large bore catheter with an ID of 1.6 mm (e.g., a 9.6 Fr single lumen Hickman). For TIVADs the volume of the reservoir should be included in the total volume. The reservoir volume depends on the TIVAD brand and is commonly ranging between 0.25 mL and 0.6 mL. The priming volume might be provided by the manufacturers for catheters which may not be trimmed.

The aim of a lock is to fill the catheter entirely. However the risk of “leakage” of the lock over time has been described and therefore it is suggested that catheters should be overfilled by approximately 15–20%. However, this extra volume can only be recommended if the locking solution does not cause adverse effects when systemically injected [21, 22]. Still a 20% extra volume is a limited volume, for example, 0.16 mL extra to a locking volume of 0.8 mL for the medial lumen of 18 G CVC. The total lock volume for this CVC lumen is 1 mL. In the literature, the reported locking volumes are significantly larger. However, in current guidelines the recommended volumes are small (twice the internal volume) and controversially also large (5–10 mL) [3, 4]. Indeed, a 5–10 mL lock volume was found in a survey among ICU nurses regarding flushing practices for short-term CVCs. Nurses reported using heparin volumes of 3 mL, 5 mL, and 10 mL [23]. Consequently, one can state that the injection of a 10 mL locking volume in a short-term CVC will result in an injection of 9 mL of heparin in the circulation without any residual effect in the catheter. On the other hand, twice the internal volume means a locking volume of, for example, 0.06 mL for a peripheral catheter, 0.8 mL for a CVC, and for 4 Fr PICC, 1.4 mL.

To avoid confusion and given the available variation in catheter length and diameter, uniform volumes for different catheter types are suggested.  Table 4 shows the calculation for a more uniform and appropriate catheter lock volume based on the internal catheter volume (or priming volume) with an added spillage of 20% and an extra volume (≤1 mL) for add-ons or extra-long catheters. Moreover a small volume of the locking solution will be left over in the syringe after manual performance of the positive pressure technique. So the extra volume might be necessary especially for nontrained healthcare workers. A uniform lock volume of 1.5 mL is recommended for all small catheters such as peripheral cannulas, midlines, PICCs, nontunnelled CVCs, and small bore tunnelled catheters. For large bore tunnelled catheters and TIVADs with a large reservoir, 2.5 mL is sufficient. If a strictly minimum lock volume is recommended, the used volume should be limited to the internal volume with, eventually, the 20% spillage.

### 4.3. Locking Regimen

For most low concentration locking solutions (e.g., a 100 U/mL heparin) the lock solution does not need to be aspirated. When the lock is renewed, the new locking solution may be instilled without aspiration or flushing with NS. Some locking solutions, which might be causing adverse events when injected into the blood circulation, must be first aspirated before renewal for example, a 5000 U/mL heparin lock. Most guidelines recommend a nonspecified “regular” flush regimen. The optimal time between two locking procedures when the catheter is not in use, is not well studied. Commonly a time period between 8 and 24 hours is suggested, although in PICCs and long-term CVCs periods of 1 week or more are also used.

For TIVADs, when accessing the port for the intermittent flushing procedure, it is recommended to flush first with a 10 mL NS, before a heparin lock. If the Huber needle is not correctly located in the reservoir, the paravenous administration of NS, in contrast to heparin, is not harmful. There is also a tendency to prolong the interval between intermittent accesses for TIVAD maintenance from monthly to every 6 to 8 weeks [24, 25] and even longer time periods are used. More research is needed to provide scientifically underpinned answers regarding the best time period to renew a lock.

Locking recommendations that are based on research and insights are summarized in Table 1.

### 4.4. Locking Solutions

#### 4.4.1. Heparin

A heparin lock was discussed back in the 1970s when IV peripheral cannulas were locked as alternative to a continuous heparin infusion to keep the cannula patent [26]. In that time, a lock of 1 mL heparin (10 U/mL) has been recommended following each IV injection of medication or every 8 hours [27]. Since then it also has become clear that the risks of heparin have to be taken in account. However, the chance of inducing an iatrogenic haemorrhage following catheter flushes is rare. The “heparin flush syndrome” has been described in one case report in which a patient developed postoperative bleeding after multiple blood samples and cardiac output determinations, resulting in two to three flushes of 500 to 1000 units per hour during a 12 hours period [28]. Heparin has a half-life of 1-2 hours [29]. Given that short half-life, a catheter lock every 6–8 hours will still be safe for the patient. Some institutions use a practical guideline to not exceed the 2000 units per 24 hours. A single dose of 900 units is approximately 16% of the heparin bolus required to acutely anticoagulate a 70 kg patient [30]. However several other risks are associated with heparin use. The risk of errors in dosage of heparin prompted the labelling of heparin as a “high alert” medication [31–34]. Heparin administration may also lead to heparin-induced thrombocytopenia and hypersensitivity to heparin. These are severe adverse effects of heparin even after exposure to small quantities of heparin from catheter flushing [35–38]. Moreover an intrinsic risk of heparin is infection because heparin stimulates* S. aureus* biofilm formation [39]. Extrinsic risks are the contamination of multiple dose vials of heparin-saline solution [40, 41] and the risks associated with breaks in the integrity of the IV system. Heparin is also associated with drug incompatibilities. Moreover, guidelines recommend the use of heparin in many different ways ranging from no heparin but NS as locking solution for peripheral cannulas to heparin at 10 to 100 U/mL for central venous catheters and TIVADs [3, 4, 42]. For all these reasons, the use of alternative locking solutions should be considered.

#### 4.4.2. Normal Saline

Discontinuation of heparin as locking solution seems to be attractive because it eliminates the risks associated with heparin while it prompt savings in nursing time, supplies, and costs for the patient and/or the institution and/or the society. Therefore the hypothesis that there is no statistical difference for locking a catheter with heparin or NS has been investigated many times in different types of catheters. A literature review was conducted to investigate level I-II evidence [43] relating to the benefits of interventions on the effectiveness of NS versus heparin as a locking solution in the prevention of malfunction. The results are summarized for the different catheter types in Table 5.

In peripheral cannulas, evidence was found for the discontinuation of the use of heparin locks in two meta-analyses in the early nineties. In these meta-analyses, studies with different heparin concentrations, ranging from 2.5 U/mL to 100 U/mL, are included [44, 45]. In a more recent meta-analysis, the evidence was confirmed that there was no statistically significant difference in duration of patency or clotting between NS versus a low concentration of heparin (10 U/mL) as a locking solution. However, the analysis showed a higher risk of clotting when locking with NS versus with a high concentration of heparin (100 U/mL) [46]. Since then, 5 randomised controlled trials (RCTs) have been published with controversial results in a wide variation in settings, populations, and variables [47–52]. For midlines no studies which investigated heparin versus NS as locking solution were found. In nontunnelled short-term CVCs, one meta-analysis was found. However, it was impossible to draw conclusions because different heparin volumes, concentrations, and administration routes (IV lock or continuous infusion or subcutaneously administered) were mixed up in the analysis [53]. Two RCTs which were published later on reported mixed results [54, 55].

Although the use of neutral and positive displacement connectors implies no heparin lock requirement, two RCTs with PICCs used the locking solution as dependent variable for occlusion rather than the connector. In the first study a positive displacement system was combined with the use of a 10 mL NS lock versus 5 mL of heparin (100 U/mL) [56]. In the second study all PICCs were connected to a neutral connector and patients were randomised to a 10 mL of NS lock, 5 mL heparin 10 U/mL, or 3 mL heparin 100 U/mL lock [57]. Not surprisingly both studies did not find a statistically significant difference in incidence of occlusion, probably due to the investigation of a superfluous use of heparin.

In tunnelled catheters, one RCT with a small sample size found no difference in nonpatency between a twice daily flush with 5 mL heparin (10 U/mL) versus a weekly flush of 9 mL NS [58]. In TIVADs one single RCT investigated the patency rates between TIVADs locked with heparin (100 U/mL) and NS. No difference in malfunction rates was found [20].

We can conclude that the use of a heparin lock at a concentration of 10 U/mL does not have any added value over the use of a NS lock in peripheral cannulas. The available scientific evidence regarding the efficacy of NS versus heparin (100 U/mL) locking in all types of catheters is weak due to the limited available methodological rigorous studies.

The use of the positive pressure technique might avoid blood influx at the catheter tip when disconnecting a syringe. This procedure is strongly associated with the knowledge and skills of the healthcare worker. To overcome this problem, supporting technologies such as valves incorporated in the catheter tip (e.g., Groshong, C.R. Bard) or at the catheter hub (e.g., PASV Technology, Navelyst Medical) have been developed. The integrated valves in PICCs, tunnelled, and port catheters are designed to avoid blood influx because the opening pressure of the valve is higher than the pressures found in the venous circulation. The valve opens only during positive pressure (injection) or negative pressure (aspiration). Needleless connectors with neutral or positive displacement have also been developed to prevent blood influx at the catheter tip. The need for a heparin lock is eliminated with the use of these valves and connectors. Therefore heparin as locking solution is no longer recommended by the manufacturers of these technologies. Few RCTs with a focus on catheter patency and locking with heparin versus NS with the help of these technologies (valves and connectors) are available. Two RCTs compared valved catheters versus nonvalved catheters. A first study from Hoffer and colleagues found a statistically significant lower occlusion rate in valved PICCs locked with NS versus nonvalved PICCs locked with heparin (10 mL, 10 U/mL) [59]. This was confirmed by a similar study in TIVADs which found a statistically significant lower incidence of malfunction in valved TIVADs locked with NS than in nonvalved TIVADs locked with heparin (10 mL, concentration not reported) [60]. Obviously, more large scale studies with different types of catheters are needed to generate evidence based knowledge regarding the value of valved technology in avoiding heparin as a catheter lock solution.

Only one RCT investigated a weekly NS lock with a positive displacement connector versus a twice weekly heparin lock with a standard cap in tunnelled catheters in the paediatric onco-hematology population. A lower patency rate was found with a NS lock and positive displacement connector than a heparin lock 200 U/mL (volume not reported) and a standard cap. No difference in total catheter dwell time was found [61]. In three RCTs the connector or catheter type (valved or not) was chosen as dependent variable for occlusion and not the type of locking solution. In two of these studies, a reduction in potential staff confusion was reported as reason for the uniform lock regimen with heparin. In the first study, patients were randomised to the TIVAD with valved catheter or TIVAD with nonvalved catheter group. All TIVADs were locked with 5 mL of heparin (50 U/mL). They found a statistically significant higher occlusion rate in the valved catheter group, despite the heparin use, than in the nonvalved group [62]. In the second study, patients with a PICC were assigned to a negative or to one of the two types of positive displacement connectors. A heparin lock (3 mL of 100 U/mL) was used in all types of connectors. A statistically significant difference between the three groups was found [63]. Finally, Khalidi and colleagues randomised patients with PICCs and midlines to a positive displacement connector or a standard cap with the use of a heparin lock (concentration and volume not reported). They found no statistically significant differences between the two groups [64]. Results from these few RCTs which investigated catheter patency combined with valved catheters and needleless connectors remain inconclusive.

Finally, three systematic reviews which included all types of catheters, with or without needleless connectors, valved or nonvalved CVCs are available. Mitchell and colleagues found weak evidence that locking with a heparin solution versus NS reduces the occlusion rate. Due to methodological concerns, no strong conclusions could be drawn [65]. These findings were confirmed in two recent systematic reviews [66, 67].

It is obvious that the available studies included different patient populations, different catheter types with different locking regimens. Moreover different malfunction definitions are used and although all of these studies had a strong methodological design a lot of them ended up with small sample sizes. All these issues might explain why mixed results are found. There is an urgent need for further well-designed studies using uniform terminology and outcome measures to investigate potential differences in malfunction rates between heparin and NS as locking solution for venous catheters. Till then, the choice to abandon heparin as locking solution is more one of weighing up advantages and disadvantages.

### 4.5. Other Anticoagulants than Heparin

Lepidurin is an anticoagulant which acts through direct thrombin inhibition. Only one small study investigated this locking solution versus heparin in IV catheters. A lepidurin (100 *μ*g) lock was not found to be superior to a heparin (100 U/mL) lock [30].

### 4.6. Thrombolytic Agents

Urokinase is a thrombolytic agent and therefore effective in the treatment of thrombotic occlusion. This fibrinolytic drug may also be used in a more prophylactic way. Moreover the use of periodic fibrinolytic therapy was also suggested in the prevention of catheter-related infectious complications [68]. Three studies have focused on the comparison between heparin and urokinase as locking solution with mixed results. Solomon and colleagues assigned patients with a tunnelled catheter to a heparin (50 U/mL, 5 mL) or urokinase (5000 U/2 mL) lock. They found that the use of twice weekly urokinase lock was not more effective in reducing infectious and thrombotic complications than a heparin lock [69]. Ray and colleagues randomised patients with a tunnelled catheter between twice daily heparin locks (10 U/mL) and a weekly urokinase lock (9000 U/1.8 mL). They found that malfunction rates were statistically significantly reduced by a urokinase lock compared to a heparin lock. This was confirmed by Dillon and colleagues who assigned paediatric patients with TIVADs and tunnelled catheters to either a heparin (100 U/mL) or urokinase (5000 U/mL, 1.8 mL) lock every two weeks [70]. No RCTs were found on the effectiveness of other thrombolytic agents such as recombinant tissue plasminogen activator or tissue plasminogen activator versus heparin as locking solution.

### 4.7. Antimicrobial and Antiseptic Lock Prophylaxis

Due to the number of manipulations over time, long-term venous catheters are prone to breaches in aseptic technique during the manipulation of the catheters. The intraluminal source of infection is associated with more prolonged dwell times [71]. Moreover microbial colonization will produce a biofilm when there is contact with a biomaterial such as the inner catheter wall [72]. An antimicrobial lock might be instilled into the catheter with a long enough dwell time to prevent colonization and biofilm formation or to eliminate the biofilm [73]. The antibiotic lock technique was first described in 1988 for the treatment of catheter-related sepsis without a tunnel or entry-site infection in tunnelled catheters in home PN patients [74]. Currently, antibiotic locks consist of a highly concentrated antimicrobial, often in combination with an anticoagulant (cefazolin, cefotaxime, ceftazidime, ciprofloxacin, daptomycin, gentamicin, linezolid, telavancin, ticarcillin-clavulanic acid, and vancomycin) [75].

A meta-analysis of trials in oncology showed weak scientific proof for effectiveness of antibiotic-based lock solutions compared to heparin in preventing CRBSI. However, in the included studies, the investigated antibiotic locks were heterogeneous (vancomycin, amikacin, and ciprofloxacin) and the outcome measurement used was nonspecific (sepsis and noncatheter related sepsis) [76]. Another systematic review in oncology patients which focused on the prevention of Gram-positive catheter-related infections in long-term CVCs showed a reduction of sepsis. The authors concluded that further research is needed to identify high risk groups most likely to benefit [77]. This is in line with the Centers for Disease Control and Prevention guidelines which state that antibiotic lock prophylaxis should be reserved for patients with long term catheters who have a history of multiple CRBSI despite optimal adherence to aseptic technique [78]. It is known that the use of an antibiotic lock may increase antimicrobial resistance and may also increase the risk of toxicity to the patient resulting from leaking or flushing of the lock solution into the systemic circulation. Moreover it was found that antibiotic treatment, similar to heparin, can stimulate biofilm adherence to the catheter surface [39, 79]. Therefore there is an urgent need for alternative nonantibiotic locks and nonheparin anticoagulants.

Nonantibiotic locks or antiseptics kill bacteria through physical effects rather than specific biochemical pathways and may not induce microbial resistance [80]. Donlan described different approaches to the control of biofilms on intravascular catheters with chelating agents, ethanol, and taurolidine [73]. Chelating agents have the potential to remove established biofilm (bacteria and fungi). Sodium citrate and ethylenediaminetetraacetic acid (EDTA) are chelating agents. EDTA is used alone or in combinations with antibiotics [80, 81].

Ethanol also has the potential to remove established biofilm (bacteria). A systematic review suggested that a prophylactic ethanol lock decreases the rates of infection and unplanned catheter removal and that ethanol lock treatment appears efficacious in combination with systemic antibiotics. However the review was based mainly on retrospective studies [82]. A recent RCT comparing heparin (50 U in 5 mL) versus 70% ethanol lock (2 hours dwell time) in hematology patients with tunnelled catheters failed to show a statistically significant reduction in central-line-associated bloodstream infection (CLABSI) rates. However the required number of included patients was not attained and therefore the lack of impact on CLABSI rates might be underestimated [83]. The use of ethanol has been associated with adverse events. Mermel and colleagues described an increased incidence of systemic side effects, breaches in the integrity of the catheter, and catheter obstruction. Further large scale RCTs to assess the safety and efficacy of ethanol lock solutions and limiting the maximum concentration of ethanol to 28% in lock solutions are suggested [84, 85]. One newly developed locking solution has reduced the ethanol concentration in the locking solution to 20% in combination with 0.01% glyceryl trinitrate and 7% citrate. This lock showed promising results in eradicating biofilm in an* in vitro* test [86].

Taurolidine, a derivative of the amino acid taurine, is an antimicrobial agent showing a broad spectrum of antimicrobial activity against both bacteria and fungi [87, 88]. A meta-analysis of 6 small studies in patients with different catheter types and taurolidine concentrations suggest that taurolidine as locking solution reduces the CRBSI incidence without obvious adverse effects and bacterial resistance [89]. Abnormal taste sensations were reported in two studies [90, 91].

Some antimicrobial and antisepticlocks are not always considered as traditional “locks.” They do not fulfill all conditions of the earlier definition that a lock is instilled for the period of time when the catheter is not in use. Antimicrobial and antiseptic locks might dwell for a limited time and a common locking solution, such as heparin, might be utilised in between.

## 5. Conclusion

Maintaining patency has always been considered essential for all types of venous catheters. Flushing with NS is important and probably the most crucial factor in the prevention of malfunction. However, evidence on flushing techniques, volumes, and regimens is lacking. Moreover, also the available scientific basis for catheter locking with heparin is weak. Hence, clinical studies with a strong methodological design and a focus on flushing and locking in relation to malfunction are urgently needed. Uniform malfunction definitions, terminology, and measurements should be used.

Meanwhile, more standardised flushing and locking volumes should be used. Flushing volumes should be at least 10 mL in order to rinse the catheter sufficiently. Locking volumes should be minimal and based on the catheter volume. A maximum of 1 mL lock volume surplus is suitable to safely fill the catheter and any add-ons. For peripheral cannulas, a high flushing and locking volume of the catheter is not needed due to the small internal volume of the catheter.

The prevention of CRBSI due to biofilm formation is an increasingly important issue. For long-term CVCs and especially in susceptible patients an antimicrobial or antisepticlock must be considered.

## Figures and Tables

**Table 1:** Flushing and locking recommendations.

Flushing recommendations	

Technique	Use a pulsatile flow when flushing
	Use a flush with 10 × 1 mL boluses with a time interval of 0.4 s between 2 boluses
	Use SAS and SBS order for the administration of mediation/fluids and blood sampling procedures

Volume	Use a 10 mL flush for all IV catheters (except for peripheral cannulas, use 5 mL)
	Use a 20 mL flush after infusion of viscous products like blood components, parenteral nutrition, and contrast media

Regimen	Flush with NS before and after administration of drugs of fluids (SAS)
	Flush with NS before and after blood sampling (SBS)

Locking recommendations	

Technique	Use the positive pressure technique when disconnecting a syringe
	Close clamps and let them closed when not in use

Volume	1.0 mL for peripheral cannulas
	1.5 mL for midlines, PICCs, nontunnelled CVCs, and small bore tunnelled catheters (≤1 mm ID)
	2.5 mL for large bore tunnelled catheters (>1 mm ID) and TIVADs (reservoir volume up to 0.6 mL, Huber needle volume not included)

Regimen	q8h–q24h for short-term catheters
	Weekly in long-term catheters
	q6w–q8w in TIVADs

**Table 2:** Internal volume of single lumen venous catheters in mL.

Catheter length (cm)	Internal diameter (mm)											
	0.5	0.6	0.7	0.8	0.9	1	1.1	1.2	1.3	1.4	1.5	1.6
10	0.02	0.03	0.04	0.05	0.06	0.08	0.09	0.11	0.13	0.15	0.18	0.20
15	0.03	0.04	0.06	0.08	0.10	0.12	0.13	0.17	0.20	0.23	0.26	0.30
20	0.04	0.06	0.08	0.10	0.13	0.16	0.17	0.23	0.27	0.31	0.35	0.40
25	0.05	0.07	0.10	0.13	0.16	0.20	0.22	0.28	0.33	0.38	0.44	0.50
30	0.06	0.08	0.12	0.15	0.19	0.24	0.26	0.34	0.40	0.46	0.53	0.60
35	0.07	0.10	0.13	0.18	0.22	0.27	0.30	0.40	0.46	0.54	0.62	0.70
40	0.08	0.11	0.15	0.20	0.25	0.31	0.35	0.45	0.53	0.62	0.71	0.80
45	0.09	0.13	0.17	0.23	0.29	0.35	0.39	0.51	0.60	0.69	0.79	0.90
50	0.10	0.14	0.19	0.25	0.32	0.39	0.43	0.57	0.66	0.77	0.88	1.00
55	0.11	0.16	0.21	0.28	0.35	0.43	0.47	0.62	0.73	0.85	0.97	1.11
60	0.12	0.17	0.23	0.30	0.38	0.47	0.52	0.68	0.80	0.92	1.06	1.21
65	0.13	0.18	0.25	0.33	0.41	0.51	0.56	0.73	0.86	1.00	1.15	1.31
70	0.14	0.20	0.27	0.35	0.45	0.55	0.60	0.79	0.93	1.08	1.24	1.41
75	0.15	0.21	0.29	0.38	0.48	0.59	0.65	0.85	0.99	1.15	1.32	1.51
80	0.16	0.23	0.31	0.40	0.51	0.63	0.69	0.90	1.06	1.23	1.41	1.61
85	0.17	0.24	0.33	0.43	0.54	0.67	0.73	0.96	1.13	1.31	1.50	1.71
90	0.18	0.25	0.35	0.45	0.57	0.71	0.78	1.02	1.19	1.38	1.59	1.81

**Table 3:** Examples of corresponding internal and outer diameters in different types of single lumen catheters.

	Internal diameter in mm											
	0.5	0.6	0.7	0.8	0.9	1	1.1	1.2	1.3	1.4	1.5	1.6
Outer diameter in French												
Ports, PUR catheters, BBraun				4.5			5			6.5		8.5
Ports, Chronoflex CRBard								6			8.5	
Ports, Silicone catheters, BBraun						6.5	8.5					10
PICC, PowerPICC CRBard						4						
Tunneled catheter, Hickman CRBard	2.7		4.2			6.6						9.6

**Table 4:** Calculation of recommended locking volumes if lock does not cause adverse effects when systemically injected.

Catheter type	Total lock volume in mL	Minimum catheter volume in mL (Approximately internal volume + 20% spillage)	Extra volume^a^
Peripheral catheters	1.0	0.04 (0.03 + 0.006)	0.9
Midline	1.5	0.5 (0.4 + 0.1)	1.0
PICC	1.5	0.7 (0.6 + 0.1)	0.8
Nontunnelled CVC	1.5	0.7 (0.6 + 0.1)	0.8
Small bore tunnelled catheter (≤1 mm ID)	1.5	0.8 (0.7 + 0.1)	0.7
Large bore tunnelled catheter (>1 mm ID)	2.5	1.6 (1.3 + 0.3)	0.9
TIVADs (reservoir volume up to 0.6 mL)	2.5	1.6 (1.3 + 0.3)	0.9

^a^Volume might be used for add-ons, Huber needle, extension set extra-long catheters, or surplus for the positive pressure technique.

**Table 5:** RCTs and meta-analyses comparing NS and heparin as locking solution.

Authors, year	Evidence regarding patency with the use of NS versus heparin	Concentration, volume of heparin	Volume of NS	Frequency	Remarks
Peripheral cannulas					

Goode et al. 1991^∗^ [44]	No statistically significant difference	2.5, 3.3, 10, 16.5, 50, 100, 132 U/mL Volume NR	NR	q8h–q24h	Small number of studies, variation in methodological quality

Peterson and Kirchhoff 1991^∗^ [45]	No statistically significant difference	1–5 mL, 10 to 100 U/mL	1–5 mL	q8h, q12h, q24h	Small number of studies, few pediatric studies, variation in methodological quality

Randolph et al. 1998^∗^ [46]	No statistically significant difference	10 U/mL Volume NR	NR	q6h, q8h, q12h	Small number of studies
	Lower patency rate in NS group	100 U/mL Volume NR	NR	q6h, q8h	Small number of studies

Gyr et al. 1995 [47]	Lower patency rate in NS group	10 U/mL Volume NR	NR	q1h–q8h	Pediatric population

LeDuc 1997 [48]	No statistically significant difference	3 mL 10 U/mL	3 mL	0.5 h–24 h	Pediatric population in emergency department setting

Niesen et al. 2003 [49]	No statistically significant difference	1 mL 10 U/mL	1 mL	q12h	Pregnant woman in emergency department setting, limited statistical power

Mok et al. 2007 [50]	No statistically significant difference	(1) 1 mL 1 U/mL (2) 1 mL 10 U/mL	1 mL	q6h, q8h	Pediatric population

White et al. 2011 [51]	No statistically significant difference	1 mL 10 U/mL	3 mL	q8h	Pediatric population, small sample size

Bertolino et al. 2012 [52]	Lower patency rate in NS group	3 mL 100 U/mL	3 mL	q12h	Large medical population

Midlines					

	No evidence available				

Nontunneled short-term CVCs					

Rabe et al. 2002 [54]	Lower patency rate in NS group	(1) 0.5 mL 5000 U/mL	(2) 0.5 mL	q48h	(3) Third arm was Vit C 200 mg/mL, 10 mL 0.5 mL of locking solution was injected after each check for blood return without proper flushing in between
Schallom et al. 2012 [55]	No statistically significant difference	3 mL 10 U/mL	10 mL	q8h	ICU and medical ward, limited statistical power, ICU and medical ward

PICCs					

Bowers et al. 2008 [56]	No statistically significant difference	5 mL 100 U/mL	10 mL	q12–24h	A positive displacement connector was used in the 3 groups, small study

Lyons and Phalen 2014 [57]	No statistically significant difference	(1) 5 mL 10 U/mL (2) 3 mL 100 U/mL	(3) 10 mL	q12h	A neutral connector was used in the 3 groups, home care setting

Tunneled catheters					

Smith et al. 1991 [58]	No statistically significant difference	5 mL 10 U/mL	9 mL	q12h q7d NS	Small sample size, paediatrics, onco-hematology patients

TIVADs					

Goossens et al. 2013 [20]	No statistically significant difference	3 mL 100 U/mL	10 mL	Heparin at discharge or q8w	Onco-hematology patients

^∗^Meta-analysis, NR: not reported.
